# Arsenic (As) and breast cancer risk

**DOI:** 10.1186/1897-4287-10-S4-A8

**Published:** 2012-12-10

**Authors:** Magdalena Muszyńska, Katarzyna Jaworska-Bieniek, Katarzyna Durda, Grzegorz Sukiennicki, Tomasz Gromowski, Anna Jakubowska, Antoni Morawski, Jan Lubiński

**Affiliations:** 1 Read-Gene SA and Pomeranian Medical University, Szczecin, Poland

## Abstract

The study was conducted to determine the correlations between serum concentration of arsenic (As) with increased or decreased predisposition to breast and ovarian cancer.

## 

All subjects analyzed in the study were divided into two groups.

The subjects from the first group were Polish women, positive for at least one of three founder mutations in BRCA1 gene dominating in Poland (5382insC, C61G, 4153delA). In the second group were Polish women, with breast cancers, unselected but negative for BRCA1 gene mutation. Persons with detected tumor were considered as cases and the others were considered as controls. In the case first group one case and two controls, and in the case of second group one case and one control, were paired regarding many criteria (e.g. age, family cancer history, cigarettes smoking), adnexectomy to achieve the maximum of similarity between them.

Arsenic was quantitatively measured in diluted serum samples by inductively coupled plasma mass spectrometry (ICP-MS) using mass spectrometer (Elan DRC-e, PerkinElmer) in DRC mode with methane as a reaction gas, for removing polyatomic interferences in measurement.

Arsenic gave statistically significant differences in the disease risk when comparing the proportion of controls and cases in a certain quartile with the same proportion in the first quartile. Individuals classified in the second (3.6 µg/l – 4.5 µg/l), third (4.5 µg/l – 5.7 µg/l) and fourth quartile (5.7 µg/l – 58 µg/l) had a significantly higher risk of breast cancer (OR=1.7, p=0.01; OR=1.25, p=0.007; OR=1.4, p=0.04, respectively) than those in the first quartile (1.1 µg/l – 3.6 µg/l) in BRCA1 gene mutation carriers. In the second group surprisingly lower risk of breast cancer was observed among individuals classified in the second quartile (2.01 µg/l – 2.97 µg/l) in comparison with fourth quartile (4.11 µg/l – 9.14 µg/l) (p=0.054, OR=2.4).

Additionally, ratio between arsenic and selenium was analyzed. For the first group the ratios with statistically significant differences between quartiles in the disease risk are shown in Table 1. In the second group there were no statistically significant differences observed.

**Table 1:** Ratios between analyzed elements and selenium among BRCA1 mutation carriers

As/Se	Cases (n=99)	Controls (n=198)	OR	p-value
0.016 - 0.044[	21(21,2%)	53(26,8%)	1.000	-
[0.044 - 0.058[	21(21,2%)	53(26,8%)	1.000	0.163776
[0.058 - 0.072[	31(31,3%)	43(21,7%)	1.819	***0.000587***
[0.072 - 0.14	26(26,3%)	49(24,7%)	1.339	***0.005561***

